# The Struggle against Cancer

**Published:** 1905-05-27

**Authors:** 


### THE STRUGGLE AGAINST CANCER.

As with syphilis, so with cancer, the greatest barrier to progress in the knowledge of the disease has been the failure to reproduce it in the lower animals. But within recent years both these disabilities have in part, at least, been removed, and syphilis has been transmitted from man to apes, while certain classes of new growth have been successfully propagated in animals. Jensen, of Copenhagen, has succeeded in infecting mice with a form of adeno-carcinoma which can be readily propagated in other creatures of the same species,[1] and attempts have been made to produce cytolytic sera for these tumours. Dr. G. H. A. Clowes,[2] experimenting with mice secured from Jensen, was able to reproduce the tumour formation in other mice with great frequency, and publishes some interesting details of his experiments. He observed that a considerable number of the mice successfully inoculated showed in course of time a spontaneous arrest and ultimate disappearance of the new growth. Observations were then made as to the curative value of the serum of mice thus spontaneously cured. Certain infected mice were injected with this so-called "immune serum," while others similarly infected were treated with similar doses of serum from normal mice, for the purpose of controlling the experiments. He found that the immune serum exercised a marked effect upon small tumours; thus, three tumours of the size of a pea disappeared entirely in four or five days, the residues consisting almost entirely of connective tissue. A larger tumour of the size of a cherry, in a mouse similarly treated with the immune serum, diminished sensibly in size, became harder, remained stationary, and after being removed by operation showed no sign of recurrence within a month. Large tumours weighing more than three or four grammes were not appreciably affected by the serum, but the terminal cachexia commonly observed in infected mice was in these much alleviated. The infected mice used for "controls" and injected with normal mouse-serum were quite unaffected by it, and in them the growths proceeded to their usual termination. Thus, all the control animals were, at the time of writing, dead, while of those treated with the immune serum the only one which had succumbed had fallen to an accidental and remote infection. Further experiments made with the sera of mice thus artificially cured showed that, while their specific activity was undoubted, it was of a minor degree than that of immune serum derived from the mice in whom the new-formation had undergone spontaneous arrest.

It cannot be denied that these results are of the highest interest, and, with limitations, encouraging, but the author very rightly enters a warning against far-reaching deductions as to the applicability of the method to therapeutic purposes. In the first place, it appears that under normal conditions the course of these tumours in mice is subject to a great range of variation. Thus, some appear within two weeks of inoculation, while others may postpone their appearance for as long as five months. Again, in some cases the tumour will grow so fast that in a week or two it equals the size of its host, while in others the fatal result may be suspended for ten or twelve weeks. It is therefore clear that where the normal variation is so great, deductions can only be drawn tentatively. Further, the proportion of spontaneous recoveries in the series was exceedingly high, reaching to from 15 to 20 per cent.—a far higher proportion than anyone has ever claimed for the disease in man; and this fact unfortunately helps to destroy the parallel between these cases and cases of human cancer. The author's views in this connection are as follows:—That, though the microscopical characteristics of the tumours remained unaltered after propagation through several generations, it is impossible from the limited data available to estimate the effect of the repeated transplantations upon their vital characters. "In any case, it appears probable that a combination of circumstances has, in the course of our experiments, led to such a modification of the original conditions as to render it possible for a relatively slight immunising factor to exert sufficient influence to turn the scale in favour of the normal protective forces of the animal. The point upon which we would lay special stress is the evidence afforded by the above experiments of the existence of immune forces antagonistic to the development of cancer." The application of these results is unfortunately limited to the possible effect for good of the serum of a patient who has himself been spontaneously cured of the disease, and such fortunate individuals are only too rare.

[1] Cent. f. Bakt., Vol. xxxiv. [2] Johns Hopkins Hosp. Bull., April 1905.

